# NEUROD2 function is dispensable for human pancreatic β cell specification

**DOI:** 10.3389/fendo.2023.1286590

**Published:** 2023-10-25

**Authors:** Perla Cota, Lama Saber, Damla Taskin, Changying Jing, Aimée Bastidas-Ponce, Matthew Vanheusden, Alireza Shahryari, Michael Sterr, Ingo Burtscher, Mostafa Bakhti, Heiko Lickert

**Affiliations:** ^1^ Institute of Diabetes and Regeneration Research, Helmholtz Munich, Neuherberg, Germany; ^2^ German Center for Diabetes Research (DZD), Neuherberg, Germany; ^3^ School of Medicine, Technical University of Munich (TUM), Munich, Germany; ^4^ Munich Medical Research School (MMRS), Ludwig Maximilian University (LMU), Munich, Germany

**Keywords:** NEUROD2, β cells, endocrinogenesis, iPSC differentiation, endocrine cells

## Abstract

**Introduction:**

The molecular programs regulating human pancreatic endocrine cell induction and fate allocation are not well deciphered. Here, we investigated the spatiotemporal expression pattern and the function of the neurogenic differentiation factor 2 (NEUROD2) during human endocrinogenesis.

**Methods:**

Using Crispr-Cas9 gene editing, we generated a reporter knock-in transcription factor (TF) knock-out human inducible pluripotent stem cell (iPSC) line in which the open reading frame of both NEUROD2 alleles are replaced by a nuclear histone 2B-Venus reporter (NEUROD2^nVenus/nVenus^).

**Results:**

We identified a transient expression of NEUROD2 mRNA and its nuclear Venus reporter activity at the stage of human endocrine progenitor formation in an iPSC differentiation model. This expression profile is similar to what was previously reported in mice, uncovering an evolutionarily conserved gene expression pattern of NEUROD2 during endocrinogenesis. In vitro differentiation of the generated homozygous NEUROD2^nVenus/nVenus^ iPSC line towards human endocrine lineages uncovered no significant impact upon the loss of NEUROD2 on endocrine cell induction. Moreover, analysis of endocrine cell specification revealed no striking changes in the generation of insulin-producing b cells and glucagon-secreting a cells upon lack of NEUROD2.

**Discussion:**

Overall, our results suggest that NEUROD2 is expendable for human b cell formation *in vitro*.

## Introduction

**Figure 1:**
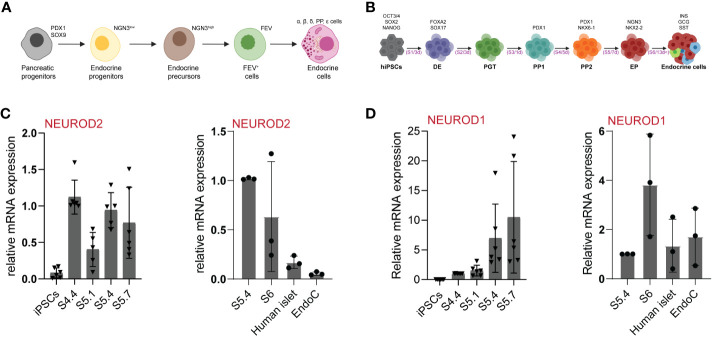
*NEUROD2* mRNA is transiently expressed during human endocrinogenesis. **(A)** Schematic picture of stepwise pancreatic endocrine lineage formation. **(B)** Schematic representation of human *in-vitro* endocrine differentiation protocol. Schemes were created with BioRender.com. **(C, D)** qPCR analysis of *NEUROD2* and *NEUROD1* expression at different stages of *in-vitro* endocrine differentiation as well as in adult primary human islets and EndoC-βH1 human β cell line.

Diabetes mellitus results from the loss or progressive dysfunction of insulin-producing pancreatic β cells that leads to hyperglycemia and severe micro- and macrovascular secondary complications. One potential strategy to treat diabetes is restoring functional β cell mass by triggering endogenous regeneration or replacing the lost β cells by islets from cadaveric donors or differentiated from human pluripotent stem cells (hPSCs) *in vitro* (1–5). The establishment of hPSC differentiation protocols into islet cells requires a thorough understanding of the mechanisms regulating endocrine lineage formation during human development (6–8). In mouse and human, endocrinogenesis is governed by differentiation of pancreatic progenitors into endocrine progenitors, which give rise to different hormone-producing endocrine cells, including α cells (glucagon^+^), β cells (insulin^+^), δ-cells (somatostatin^+^), PP cells (pancreatic polypeptide^+^) and ϵ cells (ghrelin^+^) (**Figure 1A**). This multistep lineage segregation is coordinated by variety of signaling pathways and tightly regulated by gene regulatory networks (9–11). However, it is still not well known which genetic programs orchestrate distinct endocrine cell fate decisions in humans. Thus, the functional impact of different transcription factors (TFs) on human endocrine cell induction and specification requires further investigation.

Neurogenin-3 (NEUROG3 or NGN3) is the master regulator of endocrinogenesis (12) and deletion of this TF results in endocrine cell agenesis (13). Yet, how this TF precisely regulates endocrine cell induction and specification is unclear. One possible scenario is that NGN3 controls the expression of several key endocrine lineage-specific TFs (14, 15). Among them are Neurod1 and Neurod2 that belong to the bHLH Neurod subfamily, which play important roles in tissue differentiation and lineage commitment. NEUROD1 and NEUROD2 share a high level of similarity in their bHLH domain, suggesting potential conserved functions in DNA binding and target gene activation (16–18). NEUROD2 function is important for neuronal fate specification, migration, and axonal navigation during embryonic development (19). Mice lacking *Neurod2* exhibit growth arrest, ataxia, and massive granule cell loss in the cerebellar cortex due to reduced expression of neuronal survival factors that eventually lead to early postnatal death of the knock-out animals (20). Additionally, gene polymorphisms of *Neurod2* are directly associated with neurocognitive dysfunctions, such as schizophrenia and epilepsy (21).

Despite the well-studied function of Neurod2 in neurogenesis and brain development, the function of this protein in regulating tissue differentiation in other organs, including the pancreas, has remained rudimentary. During mouse pancreas development, *Neurod2* is transiently expressed from E12.5 to E17.5, which corresponds to the peak of endocrinogenesis, while *Neurod2* is not detectable in the adult pancreas (22). Furthermore, single cell RNA-sequencing (scRNA-seq) has identified the transient expression of *Neurod2* in a subset of endocrine precursors during mouse endocrinogenesis (23). However, mice lacking *Neurod2* have shown a normal islet cell composition and morphology (22). Yet, the spatiotemporal expression pattern of NEUROD2 during human endocrine lineage formation and the function of this gene in regulating human endocrinogenesis has remained unexplored. Here, we investigated the expression and function of NEUROD2 during human endocrinogenesis by leveraging an iPSC differentiation model *in vitro*. We identified the transient expression of *NEUROD2* transcripts in human endocrine progenitors. By generating a *NEUROD2^nVenus/nVenus^* reporter iPSC line, we established a transient endocrine lineage reporter and showed that *NEUROD2* is not required for human endocrine cell induction and β cell specification.

## Materials and methods

### Cell sources

Episomal reprogrammed HMGUi001 (24) and HMGUi001-A-8 (25) iPSC line were used. All cell lines have been confirmed to be mycoplasma-free by using the Lonza MycoAlert Mycoplasma Detection Kit (Lonza, catalog no. LT07-418).

### Generation of the NEUROD2^nVenus/nVenus^ iPSC reporter cell line

The *NEUROD2* locus was targeted by homologous recombination and CRISPR/Cas9 technology using *histone 2B (H2B)-Venus-3xHA-*tag targeting vector. We cloned the sequence of the *(H2B)-Venus-3xHA-*tag into a targeting vector and used the 771 bp upstream of the *NEUROD2* start codon and 967 bp downstream of the *NEUROD2* stop codon as 5′ and 3′ homology arms (HA). A pair of gRNAs introducing dsDNA breaks 3 bp upstream of the start codon and downstream of the stop codon of the *NEUROD2* were cloned into the *pu6-sgRNA-CAG-Cas9-Venus-bpA* expression vector that allowed FACS sorting (26). This vector harboring gRNAs and Cas9 as well as the targeting vector was transfected into HMGUi001-A-8 iPSCs using the standard Lipofectamine transfection protocols. Transfected cells expressing Cas9-Venus were isolated by FACS and different colonies were picked, expanded, and tested by PCR to choose the desired clones.

### Characterization of NEUROD2^nVenus/nVenus^ iPSCs

Karyotyping of the *NEUROD2^nVenus/nVenus^* iPSC clones was executed during cell growth in a logarithmic phase. iPSC cells at passage number 29 were incubated with colcemid for 2 h, followed by trypsinization and treatment with hypotonic solution (0.075 M KCL) for 20 min and finally fixation with methanol/acetic acid (3:1). Metaphase chromosomes from *NEUROD2^nVenus/nVenus^* PSCs were classified using the standard G banding technique. The final karyotype is the average of 85% of around 20 methaphases. To test the multipotency of the *NEUROD2^nVenus/nVenus^* iPSCs, we performed a three-germ layer differentiation. Cells were differentiated in 2D monolayers to endodermal, mesodermal and ectodermal cells with the StemMACS™ Trilineage Differentiation Kit (Miltenyi Biotec, Cat# 130-115-660) according to the manufacturer’s instructions. Differentiated cells were analyzed by immunohistochemistry for endoderm (SOX17 and FOXA2), mesoderm (SNAIL1 and SM22a) and ectoderm (PAX6 and TUBB3) markers (antibody list is provided in **Table S1**). The obtained clones (HMGUi001-A-42) were registered at the European Human Pluripotent Stem Cell Registry (hPSCreg^®^): https://hpscreg.eu/cell-line/HMGUi001-A-42.

### *In vitro* differentiation of stem cell-derived islets (SC-islets)

iPSCs were cultured on 1:30 diluted Geltrex (Invitrogen, catalog no. A1413302) in StemMACS iPS-Brew medium (Miltenyi Biotec, catalog no. 130-104-368). At ~70% confluency, cultures were rinsed with 1× DPBS without Mg^2+^ and Ca^2+^ (Invitrogen, catalog no. 14190) followed by incubation with accutasse (Gibco) for 3 min at 37°C. Single cells were rinsed with iPS-Brew, and spun down at 1200 rpm for 3 min. The resulting cell pellet was suspended in iPS-Brew medium supplemented with Y-27632 (10 μM; Sigma-Aldrich, catalog no. Y0503) and the single-cell suspension was seeded at ~1.5–2×10^5^ cells per cm^2^ on Geltrex-coated surfaces for maintenance. Cultures used for 3D differentiation were seeded at 4-5 x 10^6^ cells per well in ultra-low attachment (ULA) plates, placed in a shaking platform at 60 rpm or 1 x 10^6^ cells per ml in a stirring spinner flask (ABLE corporation) stirring at 60 rpm. Cultures were fed every day with iPS-Brew medium. 3D differentiations with cells in ULA plates were started 24 h following seeding. For seeded cells in spinner flask, differentiation was started as soon as ~ 80% or more aggregates achieved a size of ~ 150 mM to ~200 mM usually 72 h after seeding.

For differentiation towards beta-like cells, the protocol from Velazco-Cruz et al., 2019 was utilized (27). Briefly, cells were first subjected to differentiate to definitive endoderm (DE) using 500 ml MCDB131 medium, a stage 1 (S1) base media for 3 days. This one supplemented with 2% bovine serum albumin (BSA) (Sigma, catalog no. 10775835001), 1X Glutamax (Gibco, catalog no. A12860-01), 0.25 mM ascorbic acid (Sigma, catalog no. A4544-25G), 1% P/S, 0.22 g glucose (MilliporeSigma; G7528), 1.23 g sodium bicarbonate (MilliporeSigma; S3817), ITS-X (Invitrogen; 51500056). S1 media was later supplemented with 100 ng/ml Activin A (R&D Systems; 338-AC) and 3 μM Chir99021 (Stemgent; 04-0004-10) the first day. Next 2 days, S1 media was supplemented with 100 ng/ml Activin A. Following that, cells were differentiated toward primitive gut tube utilizing a stage 2 (S2) media for 3 days. 500 ml of MCDB131 with 2% BSA, 1X Glutamax (Gibco, catalog no. A12860-01), 0.25 mM ascorbic acid (Sigma, catalog no. 120-14-300), 0.22 g glucose, 0.615 g sodium bicarbonate, 10 µL ITS-X, 50 ng/ml KGF (Peprotech; AF-100-19) and 1% P/S. Later, cells were further differentiated toward pancreatic endoderm using a stage 3 media (S3) for 1 day. 500 ml of MCDB131 supplemented with 1X Glutamax, 0.22 g of glucose, 0.615 g sodium bicarbonate, 2% BSA, 2.5 mL ITS-X, 0.25 mM ascorbic acid, 1% P/S, 50 ng/ml KGF, 200 nM LDN193189, (Reprocell; 040074), 500 nM PdBU (MilliporeSigma; 524390), 2 μM Retinoic Acid (MilliporeSigma; R2625), 0.25 μM Sant1 (MilliporeSigma; S4572) and 10 µM Y27632. For the formation of pancreatic progenitors, a stage 4 (S4) media was utilized for 5 days. 500 ml of MCDB131 supplemented with 1xGlutamax, 0.22 g of glucose, 0.615 g sodium bicarbonate, 2% BSA, 2.5 mL ITS-X, 0.25 mM ascorbic acid, 1% P/S, 5 ng/mL Activin A, 50 ng/mL KGF, 0.1 µM retinoic Acid, 0.25 µM SANT1, 10 µM Y27632. Next, for the formation of endocrine progenitors and hormone positive cells a stage 5 media for 7 days was added. 500 mL MCDB131 with 0.877 g sodium bicarbonate, 2% BSA, 1.8 g glucose, 2.5 mL ITS-X, 5 mL GlutaMAX, 22 mg vitamin C, 1% P/S, and 5 mg heparin (MilliporeSigma; A4544). This one was supplemented with 10 µM ALK5i II (Enzo Life Sciences; ALX-270-445-M005), 1 µM XXI (MilliporeSigma; 595790), 20 ng/mL Betacellulin (R&D Systems; 261-CE-050), 1 µM T3 (Biosciences; 64245), 0.1 µM Retinoic Acid and 0.25 µM SANT1. Finally, to allow maturation of hormone positive endocrine cells a stage 6 media (ESFM media) was then utilized for up to 35 days. 500 mL MCDB131 was supplemented with 2% BSA, 5.2 mL GlutaMAX, 1% P/S, 5 mg heparin, 0.23 g glucose, 5.2 mL MEM nonessential amino acids (Corning; 20-025-CI), 84 µg ZnSO4 (MilliporeSigma; 10883), 523 µL Trace Elements A (Corning; 25-021-CI), and 523 µL Trace Elements B (Corning; 25-022-CI).

### RNA isolation, cDNA preparation and qPCR analysis

Total RNA was extracted from cells with the miRNeasy mini kit (Qiagen). Isolated RNA was reverse transcribed using the SuperScript Vilo cDNA and cDNA synthesis kit (Life Technologies-Thermofisher Scientific). qPCR was performed using predesigned TaqMan™ probes (Life Technologies) and 20 ng of cDNA per reaction. Each reaction consisted of 4.5 µL cDNA in nuclease-free water, 5 µL TaqMan™ Advanced master mix (Life Technologies) and 0.5 µL TaqMan probe™ (Life Technologies). qPCR was performed using QuantStudio 7 Flex (Thermo Fisher Scientific). Ct-values were normalized among samples, transformed to linear expression values, normalized on reference genes and on control samples. Samples were normalized to the housekeeping genes glyceraldehyde 3-phosphate dehydrogenase (GAPDH). The list of used Taqman probes (Applied Biosystems) are provided in **Tables S1**.

### Flow cytometry

Cells undergoing endocrine differentiation were dissociated to a single cell suspension with accutase for 10-20 min at 37°C. The accutase was inactivated and discarded by washing with iPSC Brew media to later spin down at 1200 rpm for 3 min. Cells pellet was washed once with PBS and fixed with 4% paraformaldehyde for 10 min. Cells were then permeabilized with donkey blocking solution (0.1% tween-20, 10% FBS, 0.1% BSA, 0.2% Triton-X100 and 3% donkey serum). Utilizing the same permeabilization solution, cells were stained with primary antibodies (**Table S1**) for 1 hour at room temperature or at 4°C overnight. If no conjugated antibody was utilized, the protocol continued with incubation of appropriate secondary antibodies for 30 min-1 h at room temperature (**Table S1**). Cells were washed with PBS three times after antibody incubation. Flow cytometry was performed using FACS-Aria III (BD Bioscience). FACS gating was determined utilizing isotype, secondary only antibody and stained hiPSCs. FACS data were analyzed using FlowJo.

### Confocal microscopy and imaging

Cryosections rehydration started by washing 3 times with 1X PBS. For attached cell monolayer, dispersed cells at different stages were first prepared as single cell solution and plated utilizing 8 well μ-Slide (ibidi), left overnight allowing attachment to the surface and form a cell monolayer. The Cryosectioned and/or cell monolayer were next permeabilized with 0.1 M glycine and 0.2% Triton X-100 in PBS for 30 min and later blocked in blocking solution (PBS, 0.1% Tween-20, 1% donkey serum, 5% FCS) for 1 h. The sections were later incubated with the primary antibody (**Table S1**) diluted in the same blocking solution overnight at 4°C. Afterwards, 3 times washing with 1X PBS is undertaken to later incubate with secondary antibodies (**Table S1**) diluted in blocking solution. Finally, after being incubated during 2-3 h with the 2^nd^ antibody, sections were stained for DAPI (1:500 in 1X PBS) for 30 min, rinsed and washed 3x with 1X PBS and mounted using our self-made Elvanol. Images were obtained with a Leica microscope of the type DMI 6000 using LAS AF software and were analyzed and quantified using LAS AF and ImageJ software programs.

### ScRNA-seq data analysis

Throughout the analysis of scRNA-seq data, Python 3.9.16 was used, in conjunction with Scanpy (version 1.9.3) (https://github.com/theislab/scanpy) (28). Quality control and normalized scRNA-seq data were obtained from GEO (reference: GSE132188). This data captures four developmental stages of pancreatic tissue (from E12.5 to E15.5) in mouse embryonic pancreatic cells (23). Two cell types, ‘Fev+’ and ‘Ngn3 high EP’, were selected and sequestered into a subset, a Principal Component Analysis (PCA) was executed on the subset. Neighbors in the Neurod2 subset were computed using the ‘sc.pp.neighbor’ function from Scanpy, UMAP representations for the Neurod2 subset were calculated using the sc.tl.umap function of Scanpy. The Neurod2 high subset was selected based on Neurod2 expression of the ‘leiden’ attribute. Cells were categorized with a ‘Neurod2’ gene expression of less than or equal to 1 as ‘low’, and the rest as ‘high’.

For assessing differential gene expression, we utilized the tl.rank_genes_groups function’s ‘wilcoxon’ method. Differential genes were filtered using the ‘sc.tl.filter_rank_genes_groups’ function. Considering the importance of pseudo-batch data and pseudo-duplication in single-cell RNA-seq differential expression testing (29). So, we used the R package Delegate (https://github.com/cancerbits/DElegate) to strictly screen for differential genes.

Overlaps between the Differential upregulated and downregulated genes with the top 1045 of Neurod2 ChIP-Seq target genes set (30) were computed. A Venn diagram illustrating the overlaps between these three gene sets was plotted using the venn3 function.

### Statistical analysis

Comparison of three or more datasets was performed using ordinary one-way analysis of variance (ANOVA) with Bonferroni’s multiple comparison test. All statistics were performed using GraphPad Prism software 9.

## Results

To identify the expression pattern of *NEUROD2* during human endocrine lineage formation we used a 3D spinner flask *in vitro* system (**Figure 1B**) (27). qPCR analysis identified no expression of *NEUROD2* mRNA at pluripotency stage in iPSCs. We found a *NEUROD2* transcript expression peak at pancreatic progenitor stage 4 (S4) that was reduced at the beginning of endocrine induction at S5.1. However, a second peak of *NEUROD2* expression was evident at S5.4, corresponding to the peak of endocrine progenitor formation. The levels of *NEUROD2* were further reduced at S5.7 and S6, the stages corresponding to the generation of stem cell-derived hormone^+^ islet cells (SC-islets). In support of the transient expression during endocrine induction, we found no expression of *NEUROD2* mRNA in primary adult human islets and the EndoC-βH1 human β cell line (**Figure 1C**). In comparison, *NEUROD1* was expressed at the endocrine progenitor stage and the expression was even higher in S6 SC-islets, human primary islets and the EndoC-βH1 cells (**Figure 1D**). Together, these data demonstrate the transient expression of *NEUROD2* in a subset of human endocrine progenitors, similar to the expression pattern that was previously identified during mouse endocrinogenesis (23, 31).

**Figure 2:**
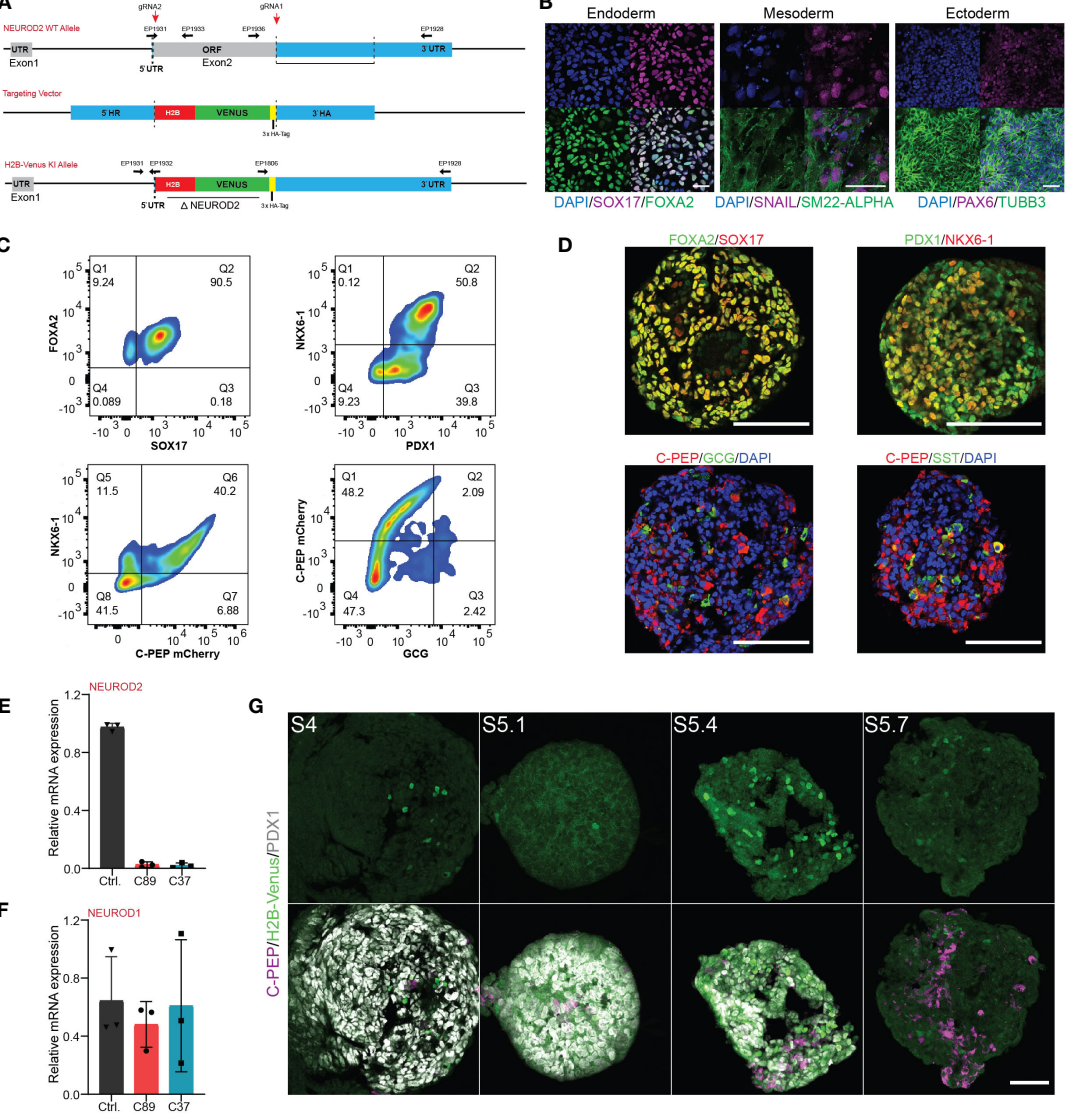
Generation and characterization of the NEUROD2 KO-H2B-Venus reporter (*NEUROD2^nVenus/nVenus^*) iPSC line. **(A)** Schematic representation of the integration of H2B-Venus sequence into the NEUROD2 exon2 locus. The targeting vector containing the H2B-Venus reporter sequence flanked by a 5′ and 3′ homology arm (HA) served as template for the repair. EPs indicate primers used for genotyping. **(B)** Representative confocal pictures of directed differentiation of *NEUROD2^nVenus/nVenus^* (C89) towards the three germ layers, endoderm: SOX17, FOXA2; mesoderm: SM22-α, SNAIL1, and ectoderm: TUBB3, PAX6. Scale bar, 50 µm. **(C)** Representative FACS plots showing the formation of definitive endoderm (FOXA2 and SOX17), pancreatic progenitors (NKX6-1 and PDX1), and endocrine cells (NKX6-1, C-PEP and GCG) confirming *in vitro* differentiation capacity of *NEUROD2^nVenus/nVenus^* (C89) cells. **(D)** Representative confocal pictures showing definitive endoderm (DE), pancreatic progenitor (PP2) and endocrine cells, validating *in vitro* differentiation potential of *NEUROD2^nVenus/nVenus^* (C89) cells. Scale bars 50 µm. **(E, F)** qPCR analysis indicating the mRNA expression levels of *NEUROD2* and NEUROD1 in differentiated control, C89 and C37 clones at S5.4. **(G)** Representative confocal pictures disclosing H2B-Venus expression at S4, S5.1, S5.4 and S5.7. Scale bar, 50 µm. Data are represented as mean ± SD.

The *NEUROD2* gene is located in the chromosome 17q12, consisting of 2 exons from which exon 1 is described as a retained intron, and exon 2 is the protein coding region. To uncover the functional impact of NEUROD2 on human endocrinogenesis, we deleted the entire open reading frame of *NEUROD2* and replaced it by a *histone 2B (H2B)-Venus-3xHA*-tag sequence in iPSCs using Crispr/Cas9 technology (**Figure 2A**). To generate the fluorescently reporter iPSC cell line lacking NEUROD2, we used the heterozygous C-peptide-mCherry reporter hiPSC line (HMGUi001-A-8), which allowed us to monitor insulin production and stem cell-derived β cell (SC-β) formation throughout *in vitro* differentiation (25) (**Figure S1A**). Genomic PCR analysis confirmed the homologous recombination at the *NEUROD2* locus resulting in generation of two homozygous *NEUROD2* knock-out *H2B-Venus* reporter gene knock-in iPSC clones (C89 and C37) (**Figure S1B**). We further confirmed the deletion of *NEUROD2* sequence and in-frame insertion of the *H2B-Venus-3xHA*-tag reporter using Sanger sequencing (**Figure S1C**). The obtained homozygous NEUROD2-KO, nuclear H2B-Venus reporter *(*NEUROD2^nVenus/nVenus^ *)* iPSC clones were negative for mycoplasma and exhibited a normal karyotype (46, XX) (**Figure S1D**). Bright-field live imaging of the *NEUROD2^nVenus/nVenus^* clones at the pluripotent stage revealed no phenotypic signs of differentiation and expression of H2B-Venus (**Figure S1E**). This was supported by immunostaining analysis indicating the expression of the pluripotency markers, SOX2 and OCT3/4 (**Figure S1F**). Furthermore, we tested for the multilineage differentiation potential of the *NEUROD2^nVenus/nVenus^* iPSC clones confirming the generation of endodermal, mesodermal and ectodermal cells (**Figure 2B**). To assess the effects of NEUROD2 TF knock-out on pancreatic endocrine lineage differentiation, we employed a six-stage (S) 3D spinner-flask differentiation protocol (**Figure 1B**) (27). FACS analysis and immunostaining revealed the successful formation of definitive endoderm (DE) at S1, pancreatic progenitor (PP2) at S4 and different pancreatic endocrine cells including SC-β (C-PEP^+^), SC-α (GCG^+^) and SC-δ cells (SST^+^) at S6 (**Figures 2C, D**). Because *NEUROD2* mRNA expression peaks at the endocrine progenitor stage, we harvested samples from the end of the endocrine induction stage at S5.4 to monitor the expression levels of *NEUROD2*. qPCR analysis detected no expression of *NEUROD2* mRNA in the *NEUROD2^nVenus/nVenus^* endocrine progenitors compared to the wild-type control cells (**Figure 2E**). Yet, all the clones expressed comparable levels of *NEUROD1* mRNA (**Figure 2F**), indicating no *NEUROD1* compensation in the absence of *NEUROD2*. Next, we analyzed the H2B-Venus reporter expression, which monitors *NEUROD2* transcriptional activity. We found emergence of Venus^+^ cells from the S4 until S5.7, with the peak of expression at S5.4 (**Figure 2G**). This result supports the expression pattern of *NEUROD2* mRNA identified by qPCR analysis. Taken together, we successfully engineered *NEUROD2^nVenus/nVenus^ reporter* knock-in knock-out iPSCs lines, which show no deficit in pancreatic endocrine lineage formation.

**Figure 3:**
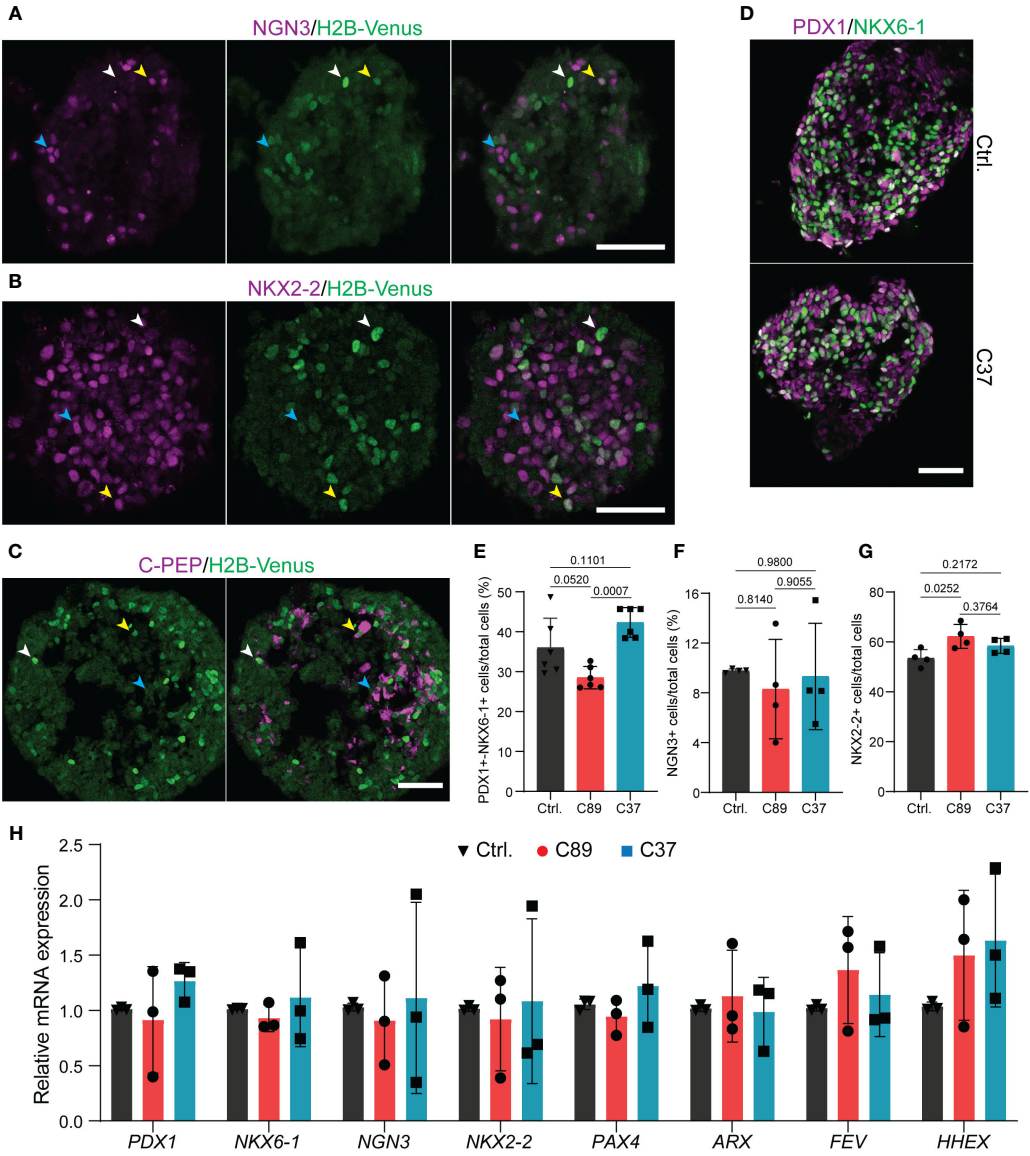
Lack of NEUROD2 has no impact on human endocrine lineage induction. **(A, B)** Representative confocal pictures showing the expression of NGN3 and NKX2-2 with H2B-Venus at S5.4 in *NEUROD2^nVenus/nVenus^* C37 clone. White arrowheads; bright Venus and low/no NGN3 or NKX2-2, blue arrowheads; high levels of NGN3 or NKX2-2 and low/no Venus, yellow arrowheads; co-expression of Venus with NGN3 or NKX2-2. Scale bar, 50 µm. **(C)** Representative confocal pictures indicating the expression of C-PEP with H2B-Venus at S5.4 in *NEUROD2^nVenus/nVenus^* C37 clone. White arrowheads; bright Venus and low C-PEP, blue arrowheads; high levels of C-PEP and low/no Venus, yellow arrowheads; co-expression of Venus with C-PEP. Scale bar, 50 µm. **(D)** Representative confocal pictures disclosing the comparable rate of formation of PP2 cells in control and C37 clone at S4.5. Scale bar, 50 µm. **(E–G)** FACS quantification of the percentage of PDX1^+^/NKX6-1^+^ at S4.5 and NGN3^+^ and NKX2-2^+^ cells at S5.4. **(H)** qPCR analysis of expression levels of *PDX1*, *NKX6*-1, *NGN3*, *NKX2-2*, *PAX4*, *ARX*, *FEV*, and *HHEX* at S5.4. All statistics have been done using one-way ANOVA. Data are represented as mean ± SD.

Next, we analyzed the NEUROD2 reporter activity in endocrine progenitors at S5.4. We found cells with bright nuclear Venus signals but expressing low or no NGN3 or NKX2-2 (**Figures 3A, B**; white arrowheads). In contrast, the major fraction of cells expressing high levels of NGN3 or NKX2-2 showed low or no Venus reporter activity (**Figures 3A, B**; blue arrowheads). A subset of cells also co-expressed medium levels of Venus with NGN3 or NKX2-2 (**Figures 3A, B**; yellow arrowheads). This data suggests that *NEUROD2* is expressed in a subset of endocrine progenitors and its expression starts after NGN3, similar to what was reported in mouse *in vivo* (23). The transient expression of NEUROD2 was further confirmed by detecting Venus reporter activity only in a subset of C-PEP^+^ cells at S5.4. A fraction of Venus^+^ cells with bright signals expressed low levels of C-PEP (white arrowheads) and a fraction of those with high C-PEP expression exhibited low Venus signal (yellow arrowheads). However, most of the C-PEP^+^ cells showed no *NEUROD2* reporter activity (blue arrowheads) (**Figure 3C**). This result indicates that at least a fraction of NEUROD2^+^ cells eventually evolved into β cell fate. Next, we explored the impact of loss of NEUROD2 function on human endocrine lineage induction. Immunostaining and FACS analysis of S4 differentiated cells revealed a comparable number of PP2 cells co-expressing PDX1 and NKX6-1 between the control and *NEUROD2^nVenus/nVenus^* cells (**Figures 3D, E**). Yet, we found a difference in the rate of PP2 cell formation between C89 and C37 clones, likely due to clone variation (**Figure 3E**). In support of this, we identified comparable numbers of NGN3^+^ progenitors between the three clones, indicating no deleterious effect in NGN3-mediated endocrine induction (**Figure 3F**). The clone C89 but not the C37 showed a slight increase in the numbers of NKX2-2^+^ cells compared to the control cells. Yet, the rate of NKX2-2^+^ cells between the two *NEUROD2^nVenus/nVenus^* cells was comparable (**Figure 3G**). To support this data, we also performed qPCR analysis at the end of endocrine induction at S5.4, which corresponds to the highest number of Venus^+^ reporter positive cells. This analysis revealed comparable levels of key pancreatic and endocrine regulatory TF mRNA expression for *PDX1, NKX6-1*, *NGN3*, *NKX2-2, PAX4, ARX* and *FEV* between the three clones (**Figure 3H**). Yet, an increased tendency but non-significant in the expression levels of the δ-cell TF *HHEX* (32) was found in the *NEUROD2^nVenus/nVenus^* cells compared to the control (**Figure 3H**). These results indicate that NEUROD2 is not required for human endocrine lineage induction.

**Figure 4:**
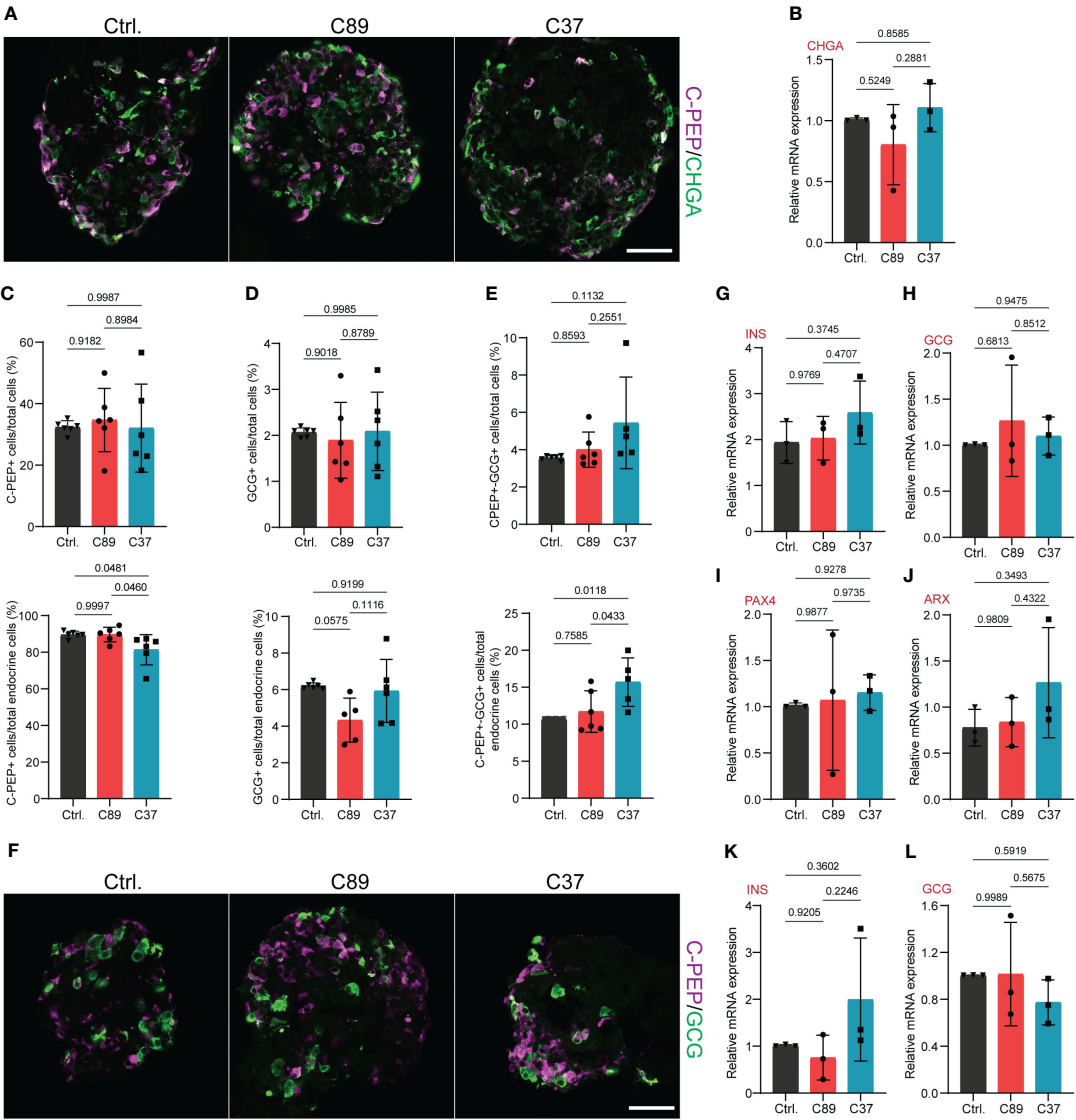
NEUROD2 function is dispensable for SC-α and SC-β cell differentiation. **(A)** Representative confocal pictures showing the C-PEP^+^ and CHGA^+^ cells at S5.7. Scale bar, 50 µm. **(B)** qPCR analysis of *CHGA* at S5.7. **(C)** FACS quantification of C-PEP^+^ cells relative to total cells (upper graph) and total endocrine cells (lower graph) at S5.7. **(D)** FACS quantification of GCG^+^ cells relative to total cells (upper graph) and total endocrine cells (lower graph) at S5.7. **(E)** FACS quantification of C-PEP^+^-GCG^+^ cells relative to total cells (upper graph) and total endocrine cells (lower graph) at S5.7. **(F)** Representative confocal pictures displaying the C-PEP^+^ and GCG^+^ cells at S5.7. Scale bar, 50 µm. **(G–J)** qPCR analysis of *INS, GCG, PAX4* and *ARX* at S5.7. **(K, L)** qPCR analysis of *INS* and *GCG* at S6. All statistics have been done using one-way ANOVA. Data are represented as mean ± SD.

To discover the function of NEUROD2 on endocrine lineage differentiation, we analyzed the formation of hormone^+^ cells at S5.7. We used immunofluorescence and stained the SC-islets for the pan-endocrine marker, CHGA, indicating a comparable number of cells expressing this protein between the three cell lines (**Figure 4A**). Furthermore, qPCR analysis of S5.7 SC-islets indicated comparable levels of *CHGA* mRNA between the three clones (**Figure 4B**). Next, we measured the rate of generation of SC-α and SC-β cells using FACS analysis. We found comparable numbers of C-PEP^+^, GCG^+^ and C-PEP^+^-GCG^+^ cells between the control and C89 clones. However, C37 cells exhibited a slightly reduced number of C-PEP^+^ cells and increased number of polyhormonal C-PEP^+^-GCG^+^ cells but no difference in the number of GCG^+^ cells compared to the control and C89 clones (**Figures 4C–E**). Immunostaining analysis indicated no apparent difference in the SC-α and SC-β cell composition between the three clones (**Figure 4F**). To further support these data, we performed qPCR analysis of S5.7 cells for endocrine cell markers. We found comparable mRNA expression levels of *INS*, *GCG*, *PAX4* and *ARX* between the three clones (**Figures 4G–J**). We also extended the qPCR analysis to the S6 SC-islets and found no changes in the expression levels of *INS* and *GCG* between the three clones (**Figures 4K, L**). This analysis demonstrates that NEUROD2 function is not required for SC-α and SC-β cell specification.

**Figure 5:**
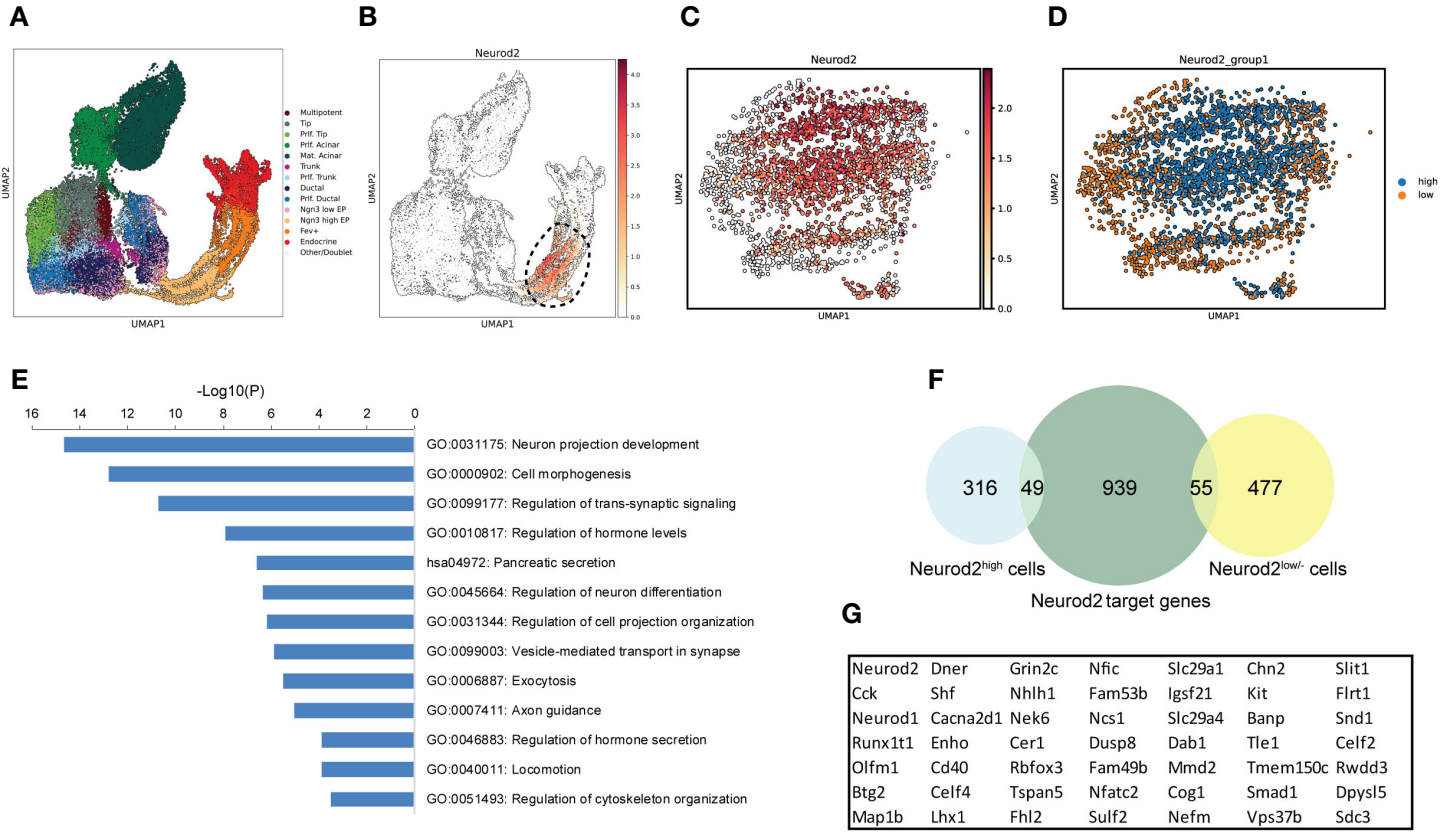
Profiling of *Neurod2*-expressing cells during endocrinogenesis *in vivo*. **(A)** UMAP plot of single cells from embryonic (E12.5-E15.5) pancreatic cells from Bastidas-Ponce et al., 2019. **(B)** UMAP plot displaying the distribution of cells considered as *Neurod2* ^+^ cells. **(C)** UMAP plot showing the different expression levels of *Neurod2*. **(D)** UMAP plot indicating two clusters based on *Neurod2* expression levels (Neurod2^high^ and Neurod2^low/-^). **(E)** Pathway enrichment analysis of the 365 upregulated genes in Neurod2^high^ cluster compared to Neurod2^low/-^ cells. Selected terms are presented. The whole list of the pathway enrichment analysis is provided in **Table S2**. **(F)** Venn diagram showing the overlapping between the upregulated and downregulated genes in Neurod2^high^ cluster with the Neurod2 potential target genes from the Bayam et al., 2015. **(G)** The list of 49 Neurod2 potential target genes upregulated in Neurod2^high^ cells.

We next explored the molecular signatures that are likely associated with NEUROD2 function during endocrine lineage formation. Because there are no available human datasets highly enriched for endocrine progenitors expressing *NEUROD2*, we leverage a scRNA-seq of mouse endocrinogenesis *in vivo*, which contains a high number of endocrine progenitors (**Figure 5A**). We detected expression of *Neurod2* mainly in endocrine progenitors and in a fraction of Fev^+^ cells (**Figure 5B**). We then selected all the cells at the time of *Neurod2* expression along the pseudotime (circle in **Figure 5B**) and reclustered them into two populations expressing high levels of *Neurod2* (Neurod2^high^) and low or no levels of this TF (Neurod2^low/-^) (**Figures 5C, D**). Differential gene expression analysis revealed 365 upregulated and 532 downregulated genes when comparing the Neurod2^high^ cluster to the Neurod2^low/-^ cells (**Table S2**). We performed pathway enrichment analysis of the upregulated genes using the Metascape annotation and analysis resource (33). Most of the upregulated genes were involved in neuronal cell development and differentiation, cell projection and morphogenesis, hormone secretion, exocytosis and cytoskeleton organization (**Figure 5E**). Among these, we found upregulation of several genes associated with axonal guidance and growth including *Plxna3*, *Efna3*, *Fez1*, *Dpysl5*, *Emb*, *Arc*, *Flrt1*, *Dab1*, *Olfm1* and *Shroom3*. Furthermore, several genes involved in cell migration and cytoskeletal remodeling including *Kit*, *Pgf*, *Slit1*, *Sdc3*, *Map1b*, *Pak3*, *Arhgef2*, *Fermt2*, *Mapre3*, *Cyfip2* and *Scin* were upregulated in Neurod2^high^ cells (**Table S2**). This analysis demonstrates the expression of *Neurod2* at the stage, in which significant changes in cell dynamics occur, and it likely corresponds to the endocrine cell egression stage during islet cell morphogenesis. To investigate the possible interlink between Neurod2 function and the highly expressed genes in Neurod2^high^ cells we used a previously reported ChIP-Seq (chromatin-immunoprecipitation and sequencing) dataset of Neurod2 potential target genes in lineages of cortical projection neurons during neurogenesis (30). Comparing the upregulated genes in Neurod2^high^ cells with the top 1043 Neurod2 potential targets resulted in identification of 49 overlapped genes (**Figure 5F**; **Table S2**). Interestingly, several of these genes including *Kit*, *Slit1*, *Sdc3*, *Map1b*, *Dab1*, *Olfm1*, *Dpysl5* and *Flrt1* are involved in morphogenetic processes (**Figure 5G**). Among them, Slit1 belongs to Slit family of secreted extracellular matrix proteins, which have been recently reported to regulate endocrine cell morphogenesis during mouse endocrinogenesis (34, 35). Therefore, this analysis predicts a possible function of Neurod2 in regulating programs that coordinate endocrine cell morphogenesis.

## Discussion

The gene regulatory networks governing human pancreatic endocrine lineage during embryonic development are still not well explored. Here, we analyzed the spatiotemporal expression pattern of *NEUROD2* and the functional impact of its deletion during human endocrinogenesis by combining Crispr/Cas9 technology and iPSC differentiation system *in vitro*. We successfully generated an iPSC reporter line in which the coding sequence of *NEUROD2* was replaced by a *H2B-Venus* sequence that allowed monitoring the *NEUROD2* transcriptional activity, while deleting the *NEUROD2* gene. The obtained homozygous clones were pluripotent and successfully differentiated toward all three embryonic lineages. Because *NEUROD2* is expressed in many other cell types including neurons (20), the generated line is a valuable tool to study the dynamic expression pattern and functional role of NEUROD2 in the development of other human cell types, such as during neurogenesis and intestinal endocrinogenesis. Furthermore, the expression of H2B-Venus reporter in the *NEUROD2^nVenus/nVenus^* cells enables having a short lineage tracing tool and allows the specific isolation of *NEUROD2*-expressing cells for further analysis such a gene profiling and proteomics.

Our data revealed no significant impact of loss of NEUROD2 on endocrine cells induction. This finding was not surprising as the expression of *NEUROD2* initiates after NGN3 induction (23). Additionally, the levels of *NEUROD2* has been reduced in mice lacking Nng3 (14), further suggesting the function of NEUROD2 downstream of Ngn3. Our analysis also did not show striking changes in the rate of formation of SC-α and SC-β cells as the two major types of hormone-producing cells in the pancreas. This finding is aligned well with the previous study in which lack of NEUROD2 did not show significant impact on mouse β cell differentiation (31). However, our previous scRNA-seq analysis predicted a potential relationship between endocrine precursors expressing Neurod2 with a β cell fate during mouse endocrinogenesis (23). Therefore, it is possible that the NEUROD2 function is important to regulate only a subset of β cell-specific programs during development and it is not crucial for their overall differentiation. Additionally, the possible functional impact of NEUROD2 loss on β cell formation might have been masked by the function on NEUROD1. Although we did not find increased *NEUROD1* expression upon lack of *NEUROD2*, the high expression levels of NEUROD1 might be sufficient to overcome the possible phenotype resulting from NEUROD2 deletion. In the future, deep gene-regulatory network studies including single-cell transcriptomics and epigenomics (36, 37) combined with NEUROD2 target gene profiling in human endocrine lineage, as has been reported for NGN3 (15), may reveal more subtle phenotypes and functions of NEUROD2 in flow sorted cells during human β cell development. Moreover, due to the increased expression levels of genes associated with hormone secretion and exocytosis in Neurod2^high^ cells, the possible impacts of NEUROD2 on β cell maturation and function still needs to be investigated.

Previous studies have shown the transient and restricted expression of *Neurod2* in a subset of murine endocrine progenitors (23, 31). Similarly, our data also indicated an increased expression of *NEUROD2* mRNA at the peak of NGN3^+^ endocrine progenitor formation during human endocrinogenesis. This evolutionarily conserved transient expression pattern suggests that NEUROD2 likely functions only for a short time to regulate molecular programs during endocrinogenesis. Importantly, this time window corresponds to the major morphogenetic events initiated by Ngn3 activity resulting in endocrine cell egression from the pancreatic epithelium to form proto-islets (38, 39). In line with this notion, analysis of mouse scRNA-seq data revealed a positive correlation between Neurod2 expression and expression of several genes involved in endocrine cell dynamics such as those regulating cell polarity, migration and cytoskeletal remodeling. Interestingly, our analysis also predicted possible regulatory function of Neurod2 in the expression of some of these genes. This finding suggests that NEUROD2 might be partially involved in orchestrating endocrine cell morphogenetic programs. Future studies using 3D organoids combined with single cell live imaging (40–42) should address whether NEUROD2 impacts human endocrine cell egression and islet cell morphogenesis. Interestingly, we also found upregulation of genes associated with axonal guidance and growth in Neurod2^high^ cells. Considering NEUROD2’s role in coordinating synaptic innervation, its impact on neuronal excitability, and synaptic function (43, 44), it prompts the question of whether this protein plays a role in the establishment of pancreatic islet innervation.

In summary, our data provides the expression pattern analysis of NEUROD2 during human pancreas development and uncovers the dispensable function of this TF in regulating SC-α and SC-β cell differentiation in humans. The current differentiation protocols are designed to generate mainly SC-β cells. Therefore, future analysis of the generated clones using protocols to optimally generate other endocrine cells including SC-δ and SC-ϵ cells might precisely reveal whether NEUROD2 function regulates the proper segregation of other endocrine cell types in humans.

## Data availability statement

The original contributions presented in the study are included in the article/**Supplementary Material**. Further inquiries can be directed to the corresponding authors.

## Ethics statement

The studies involving humans were approved by Ethics Committee of the Medical Faculty of the Eberhard Karls University, Tübingen. The studies were conducted in accordance with the local legislation and institutional requirements. Written informed consent for participation was not required from the participants or the participants’ legal guardians/next of kin because the used iPSC line was derived from an original clone, for which participants gave informed consent prior to entry into the original study (Wang et al., 2018). Therefore, such consent was not required for this study again.

## Author contributions

PC: Formal Analysis, Investigation, Visualization, Writing – original draft, Writing – review & editing, Methodology, Validation. LS: Formal Analysis, Investigation, Methodology, Validation, Visualization, Writing – review & editing. DT: Investigation, Methodology, Writing – review & editing. CJ: Writing – review & editing, Data curation, Software. AB-P: Methodology, Writing – review & editing. MV: Writing – review & editing, Methodology. AS: Writing – review & editing, Methodology. MS: Supervision, Writing – review & editing, Software. IB: Supervision, Writing – review & editing, Methodology. MB: Writing – review & editing, Conceptualization, Data curation, Formal Analysis, Funding acquisition, Investigation, Project administration, Supervision, Visualization, Writing – original draft. HL: Writing – review & editing, Conceptualization, Funding acquisition, Resources, Supervision.

## Supplementary material

Supplementary Figure 1 Generation and characterization of the *NEUROD2^nVenus/nVenus^* reporter iPSC line. **(A)** Scheme of targeting of the heterozygous C-peptide-mCherry reporter hiPSC line (HMGUi001-A-8) to generate *NEUROD2^nVenus/nVenus^* reporter iPSC line. **(B)** PCR analyses for genotyping of the control and *NEUROD2^nVenus/nVenus^* clone. Genotyping of C89 is presented. **(C)** Representative Sanger sequencing picture depicting the correct integration of H2B-Venus sequence into NEUROD2 locus in C89. **(D)** Clones C89 and C37 are characterized by a normal female karyotype (46, XX). **(E)** iPSC colony integrity of clones C89. f) SOX2 and OCT3/4 expression in clones C89. Scale bar, 80 µm.

Supplementary Table 1 The lists of used primers for genotyping PCR, probes for qPCR, and primary and secondary antibodies used for FACS and immunostaining are provided.

Supplementary Table 2 Differential expressed genes in Neurod2^high^ and Neurod2^low/-^ cells and the corresponding pathway analyses of the upregulated list. The list of overlapped genes between the list of upregulated or downregulated genes in Neurod2^high^ cells and the list of Neurod2 target genes from Bayam et al., 2015.
