# Supplementary figures and images for: NEUROD2 function is dispensable for human pancreatic β cell specification
Source: Front Endocrinol (Lausanne). 2023 Oct 25;14:1286590. doi: 10.3389/fendo.2023.1286590 (PMC10634430; doi:10.3389/fendo.2023.1286590)

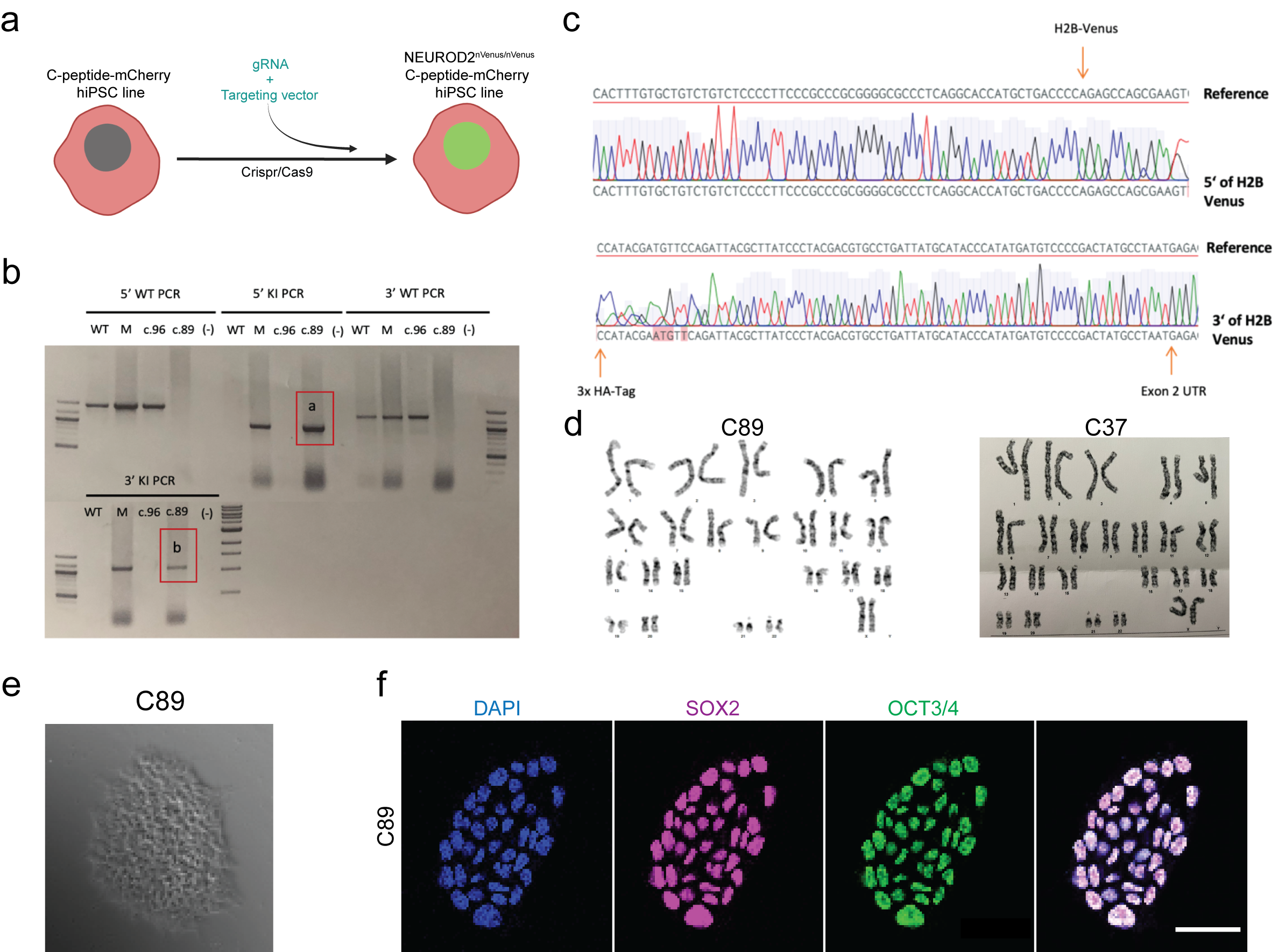

Supplement: Supplementary Figure 1 — Generation and characterization of the NEUROD2nVenus/nVenus reporter iPSC line. (A) Scheme of targeting of the heterozygous C-peptide-mCherry reporter hiPSC line (HMGUi001-A-8) to generate NEUROD2nVenus/nVenus reporter iPSC line. (B) PCR analyses for genotyping of the control and NEUROD2nVenus/nVenus clone. Genotyping of C89 is presented. (C) Representative Sanger sequencing picture depicting the correct integration of H2B-Venus sequence into NEUROD2 locus in C89. (D) Clones C89 and C37 are characterized by a normal female karyotype (46, XX). (E) iPSC colony integrity of clones C89. f) SOX2 and OCT3/4 expression in clones C89. Scale bar, 80 µm. [file Image_1.tif]
